# Bone Morphogenetic Protein-8B Levels at Birth and in the First Year of Life: Relation to Metabolic-Endocrine Variables and Brown Adipose Tissue Activity

**DOI:** 10.3389/fped.2022.869581

**Published:** 2022-03-24

**Authors:** Cristina Garcia-Beltran, Joan Villarroya, Cristina Plou, Aleix Gavaldà-Navarro, Paula Casano, Rubén Cereijo, Francis de Zegher, Abel López-Bermejo, Lourdes Ibáñez, Francesc Villarroya

**Affiliations:** ^1^Endocrinology Department, Sant Joan de Déu Research Institute, University of Barcelona, Barcelona, Spain; ^2^Centro de Investigación Biomédica en Red de Diabetes y Enfermedades Metabólicas Asociadas, ISCIII, Madrid, Spain; ^3^Department of Biochemistry and Molecular Biomedicine, Institute of Biomedicine, Sant Joan de Déu Research Institute, University of Barcelona, Barcelona, Spain; ^4^Centro de Investigación Biomédica en Red Fisiopatología de la Obesidad y Nutrición, ISCIII, Madrid, Spain; ^5^Department of Development and Regeneration, KU Leuven, Leuven, Belgium; ^6^Pediatric Endocrinology Research Group, Dr. Josep Trueta Hospital, Girona Biomedical Research Institute (IDIBGI), Girona, Spain

**Keywords:** bone morphogenetic protein-8B, brown adipose tissue, neonate, perinatal metabolism, adipokine

## Abstract

**Objective:**

Bone morphogenetic protein-8B (BMP8B) is an adipokine produced by brown adipose tissue (BAT) contributing to thermoregulation and metabolic homeostasis in rodent models. In humans, BAT activity is particularly relevant in newborns and young infants. We assessed BMP8B levels and their relationship with BAT activity and endocrine-metabolic parameters in young infants to ascertain its potentiality as biomarker in early life.

**Materials and Methods:**

BMP8B concentrations were assessed longitudinally by ELISA in a cohort of 27 girls and 23 boys at birth, and at age 4 and 12 months, together with adiposity parameters (DXA), and circulating endocrine-metabolic variables. BAT activity was measured by infrared thermography. BMP8B gene expression (qRT-PCR) was determined in BAT, white fat, and liver samples from neonatal necropsies, and in placenta and cord blood.

**Results:**

BMP8B levels were high at birth, particularly in boys (*P* = 0.04 vs. girls), declined progressively, and remained well above those in healthy adults and pregnant women at age 1 year (*P* < 0.05 and *P* < 0.001, respectively). Neonatal BMP8B transcript levels were higher in BAT than in white fat, liver and cord blood. Circulating BMP8B levels during the first year of life marginally correlated with bone mineral density and gains in lean mass.

**Conclusion:**

BMP8B levels are high at birth and decline progressively over the first year of life remaining above adult levels. Although changes in BMP8B concentrations overall reflect those in BAT activity during development, BMP8B levels are unlikely to be useful to predict individual variations in endocrine-metabolic status and BAT activity in healthy young infants.

## Introduction

Brown adipose tissue (BAT) possesses marked metabolic and thermogenic properties. In humans, BAT is especially abundant in newborns (1). BAT secretes regulatory proteins, termed brown adipokines or batokines, which hold autocrine, paracrine and endocrine attributes (2). The identification of batokines that could serve as circulating biomarkers of BAT activity could be useful in clinical practice. In this regard, we recently showed that BAT activity and the levels of the batokine C-X-C motif chemokine ligand 14 (CXCL14) -a purported surrogate of BAT activity- are sex-specific in the first year of life (3).

Bone morphogenetic protein-8B (BMP8B) is a member of the transforming growth factor β superfamily (TGF-β), a large group of regulatory factors involved in embryo development and adult tissue homeostasis. Experimental data in adult rodent models has revealed a thermogenic role for BMP8B, which has been demonstrated to be produced in BAT as a brown adipokine and to induce thermogenesis by acting both peripherally and centrally (4, 5). Other data in experimental models have shown that, despite BMP8B expression is minimal in healthy liver, hepatic BMP8B expression is increased under conditions of non-alcoholic steatohepatitis (6, 7). Recently, blood BMP8B levels have been found to be positively associated with body temperature and thermal stress-induced BAT activity in human neonates, thus indicating that BMP8B may be relevant for thermoregulation in human early life (8). It is known that BAT is highly present in the first years of human postnatal development (1), and considering that BAT is a major site of BMP8B release in rodent models (4), it could be hypothesized that BAT may be a major source of circulating BMP8B in human neonates. Thus, BMP8B levels might be a biomarker indicative of the extent of BAT activity in early life and, considering the association of BAT activity and a healthy metabolic status in adults (9), BMP8B could also reflect postnatal metabolic status.

Here, we determined for the first time the longitudinal outcome of circulating BMP8B levels over the first year of life of humans and analyzed their association with endocrine and metabolic parameters as well as with BAT activity.

## Methods

### Study Population

The study cohort consisted of 50 infants (27 girls and 23 boys) who were enrolled prenatally during the customary third trimester visit among those Caucasian pregnant mothers (age, 34.0 ± 0.7 years) consecutively seen in the outpatient clinic of Hospital Sant Joan de Déu and Hospital de Sant Boi – Parc Sanitari Sant Joan de Déu (Barcelona, Spain) (Supplementary Figure 1). Forty-three out of those 50 infants (23 girls and 20 boys) had previously participated in a longitudinal study assessing BAT activity and the circulating levels of CXCL14 in the first year of life (3).

Specific inclusion and exclusion criteria have been previously described in detail (3). Briefly, the inclusion criteria were: singleton uncomplicated pregnancies at term (37–42 weeks), exclusive breastfeeding or formula-feeding in the first 4 months, postnatal follow-up completed (at 15 days, 4 and 12 months) and written informed consent. Exclusion criteria were maternal disease, alcohol or drug abuse, congenital malformations and complications at birth. Birth weight was not considered as inclusion or exclusion criterium; accordingly, the study population included infants with a wide range of birth weight *Z*-scores (between −2.9 and +1.0).

Circulating BMP8B was measured in a subset of infants who had spare serum sample available at birth (22 girls and 20 boys), and in the whole study cohort (27 girls and 23 boys) at age 4 and 12 months. Serum BMP8B was also measured in 42 mothers of those infants during the third trimester of pregnancy (age, 34.0 ± 0.7 years) (Supplementary Figure 1). In addition, serum BMP8B concentrations were measured cross-sectionally in apparently healthy adult women (*N* = 15; age, 22.2 ± 0.8 years) and men (*N* = 11; age, 21.9 ± 0.6 years) and in 11 healthy newborns (*N* = 6 girls and *N* = 5 boys; birth weight, 3.3 ± 0.1 kg) sampled at the postnatal age of 36 h (10).

Bone morphogenetic protein-8B mRNA expression levels were assessed in: (a) dorso-interscapular BAT (*N* = 5), omental/perirenal white adipose tissue (WAT, *N* = 5) and liver (*N* = 4) post-mortem samples obtained on occasion of autopsies (2–3 h after the death) of Caucasian newborns with a gestational age of 22–36 weeks who survived, at most, 3 days post-partum, as described previously (11) (see Supplementary Table 1 for details), (b) placental samples from healthy women (*N* = 3) with uncomplicated pregnancies undergoing cesarean section at term, as previously described (12), (c) whole cord blood (*N* = 4; 2 girls, 2 boys) obtained from healthy neonates randomly selected among those composing the cohort reported above.

### Assessments

Maternal data were retrieved from the hospital clinical records. Gestational age was estimated based on the last menses and validated by first-trimester ultrasound. Weight and length were measured immediately after delivery, and again at age 4 and 12 months, and Z-scores were derived using regional normative data (13).

Maternal venous samples were obtained during the third trimester of gestation (gestational age, 35.3 ± 0.4 weeks). Infant blood samples were obtained at birth [from the umbilical cord before placenta separation (14)] and in the fasting state at age 4 and 12 months. Whole blood collected in EDTA tubes was used for RNA extraction. The serum fraction was also obtained and stored at −80°C until analysis.

Serum glucose, insulin, insulin-like growth factor (IGF)-I, high-molecular-weight (HMW) adiponectin and CXCL14 were assessed as reported (3). Serum BMP8B levels were determined with a specific human enzyme-linked immunosorbent assay kit (MBS944757, MyBioSource, San Diego, CA, United States; sensitivity: 2.34 pg/ml; intra-assay coefficient of variation (CV): <8%; inter-assay CV: <10%). Body composition was assessed by dual-energy X-ray absorptiometry (DXA) with a Lunar Prodigy and Lunar software (version 3.4/3.5; Lunar Corp., Madison, WI, United States) adapted to for infants (14).

As previously detailed (3), BAT activity at age 12 months was estimated through the infrared thermography-based measurement of the skin temperature overlying BAT depots. The parameters assessed included the maximal temperature at the posterior cervical (T_PCR_) and supraclavicular (T_SCR_) regions, and the extent of active BAT in these regions (Area_PCR_ and Area_SCR_).

RNA was extracted from tissues using an affinity-based method (NucleoSpin, MACHEREY-NAGEL, Germany) and from cord blood using TriPure (Roche Diagnostics, Indianapolis, IN, United States). BMP8B transcript levels were determined by qRT-PCR using TaqMan technology (Thermo Fisher Scientific, Waltham, MA, United States). 0.5 μg RNA were retrotranscribed using random hexamer primers (Thermo Fisher Scientific, Waltham, MA, United States). For qRT-PCR, the BMP8B TaqMan Gene Expression assay probe Hs01629120 was used, with reaction mixtures containing 1 μL cDNA, 10 μL TaqMan Universal PCR Master Mix (Thermo Fisher Scientific, Waltham, MA, United States), 250 nM probes and 900 nM of primers from the Assays-on-Demand Gene Expression Assay Mix (Thermo Fisher Scientific, Waltham, MA, United States). The 18S rRNA transcript (Hs99999901) was measured as housekeeping reference gene. The mRNA level of BMP8B in each sample was normalized to that of the reference control using the comparative (2^–ΔCT^) method.

### Statics and Ethics

Data were analyzed using the SPSS version 27.0 (SPSS software, IBM, Armonk, NY, United States). Results are shown as mean ± SEM. Variables with normal distribution were compared with two-tailed Student’s *t*-test. Chi-square test was used to compare qualitative variables. Correlation and stepwise multi-regression analysis were used to study associations between circulating BMP8B levels and the assessed variables. Covariance analysis was used to adjust for ponderal index and breastfeeding. A *P*-value < 0.05 was considered statistically significant.

The study was approved by the Institutional Review Board of University of Barcelona, Sant Joan de Déu University Hospital; informed written consent was obtained from mothers at the time of recruitment.

## Results

The anthropometric data in the mothers and the longitudinal infant data split by sex are summarized in Table 1. The infants’ results confirmed the previously reported sex-specificity of lean mass, CXCL14 and BAT activity (3).

**TABLE 1:** Maternal data and longitudinal infant data at birth and at age 4 and 12 months.

	At birth		At 4 months		At 12 months		Δ 0–12 months	
	Girls (*N* = 27)	Boys (*N* = 23)	Girls (*N* = 27)	Boys (*N* = 23)	Girls (*N* = 27)	Boys (*N* = 23)	Girls (*N* = 27)	Boys (*N* = 23)
**Mothers**								
Age (years)	33.5 ± 0.8	34.7 ± 1.1	–	–	–	–	–	–
Pre-gestational weight (kg)	64.7 ± 2.3	65.5 ± 3.9	–	–	–	–	–	–
Pre-gestational BMI (kg/m^2^)	25.0 ± 0.9	25.2 ± 1.4	–	–	–	–	–	–
Primiparous (*N*)	16	12	–	–	–	–	–	–
Cesarean section (*N*)	5	7	–	–	–	–	–	–
Smoking (*N*)	1	3	–	–	–	–	–	–
**Infants**								
**Anthropometry**								
Gestational age (weeks)	38.8 ± 0.3	38.4 ± 0.3	–	–	–	–	–	–
Breastfeeding (*N*, %)	18 (67%)	14 (61%)	–	–	–	–	–	–
Weight (kg)	2.7 ± 0.1	2.8 ± 0.1	**5.5 ± 0.1**	6.2 ± 0.2***	**8.7 ± 0.2**	9.5 ± 0.2**	**6.0 ± 0.1**	6.7 ± 0.1*
Weight *Z*-score	−1.1 ± 0.2	−1.1 ± 0.3	−1.9 ± 0.2	−1.2 ± 0.2	−0.8 ± 0.2	−0.8 ± 0.2	0.3 ± 0.2	0.2 ± 0.3
Length (cm)	48.0 ± 0.4	48.4 ± 0.6	**59.3 ± 0.8**	61.5 ± 0.8**	71.5 ± 0.9	74.1 ± 1.0	23.5 ± 0.9	25.7 ± 0.3
Length *Z*-score	−0.6 ± 0.2	−0.5 ± 0.3	−1.8 ± 0.3	−1.3 ± 0.3	−1.0 ± 0.4	−0.5 ± 0.4	−0.5 ± 0.4	−0.2 ± 0.5
BMI (kg/m^2^)	11.7 ± 0.2	11.9 ± 0.3	**15.8 ± 0.3**	16.8 ± 0.3**	**16.9 ± 0.4**	17.4 ± 0.5**	5.1 ± 0.4	5.5 ± 0.6
BMI Z-score	−0.7 ± 0.2	−0.7 ± 0.3	−0.5 ± 0.2	−0.4 ± 0.3	−0.3 ± 0.3	0.0 ± 0.3	0.4 ± 0.3	0.7 ± 0.4
**Body composition (DXA)^a^**								
Bone mineral density (g/cm^2^)	0.24 ± 0.01	0.24 ± 0.01	0.27 ± 0.01	0.27 ± 0.01	0.34 ± 0.01	0.35 ± 0.01	0.10 ± 0.01	0.11 ± 0.01
Fat mass (kg)	0.57 ± 0.03	0.57 ± 0.04	2.17 ± 0.07	2.24 ± 0.10	3.29 ± 0.14	3.53 ± 0.28	2.73 ± 0.16	2.96 ± 0.35
Abdominal fat (kg)	0.02 ± 0.00	0.02 ± 0.00	0.13 ± 0.01	0.13 ± 0.01	0.18 ± 0.01	0.16 ± 0.01	0.16 ± 0.02	0.14 ± 0.02
Lean mass (kg)	2.51 ± 0.09	2.66 ± 0.11	**3.94 ± 0.01**	4.50 ± 0.01***	**6.13 ± 0.14**	7.00 ± 0.5***	**3.62 ± 0.13**	4.33 ± 0.14***
Fat-to-lean mass ratio	0.23 ± 0.01	0.21 ± 0.01	0.55 ± 0.02	0.50 ± 0.02	0.54 ± 0.02	0.51 ± 0.04	0.31 ± 0.03	0.30 ± 0.06
**Endocrine-metabolic variables**								
Glucose (mmol/l)	−	−	4.9 ± 0.1	4.8 ± 0.0	4.5 ± 0.1	4.3 ± 0.2	−	−
Insulin (pmol/l)	−	−	29.5 ± 5.2	27.4 ± 5.4	30.9 ± 7.3	19.6 ± 5.6	−	−
IGF-I (μg/l)	−	−	34.6 ± 2.8	37.9 ± 3.7	56.2 ± 4.0	46.9 ± 5.3	−	−
HMW adiponectin (mg/l)^b^	−	−	27.6 ± 2.3	29.4 ± 1.9	13.1 ± 0.7	15.0 ± 1.9	−	−
CXCL14 (ng/l)^b^	−	−	2.8 ± 0.5	3.4 ± 0.4	**4.8 ± 0.5**	3.2 ± 0.5*	−	−
**Assessment of BAT activity^c^**								
T_PCR_ (°C)	−	−	−	−	35.7 ± 0.2	35.6 ± 0.2	−	−
T_PCR_ – T_SK_ (°C)	−	−	−	−	**1.3 ± 0.1**	0.9 ± 0.1*	−	−
Area_PCR_ (px^2^)	−	−	−	−	**1104.6 ± 147.9**	698.0 ± 107.8*	−	−
T_SCR_ (°C)	−	−	−	−	35.6 ± 0.1	35.8 ± 0.1	−	−
T_SCR_ – T_SK_ (°C)	−	−	−	−	1.2 ± 0.1	1.2 ± 0.1	−	−
Area_SCR_ (px^2^)	−	−			550.2 ± 78.3	683.5 ± 121.0	−	−

Statistically significant values are in bold. Values are mean ± SEM. BMI, body mass index; DXA, dual X-ray absorptiometry; IGF-I, insulin-like growth factor-I; HMW, high-molecular-weight; CXCL14, C-X-C motif chemokine ligand 14; BAT, brown adipose tissue; PCR, posterior cervical region; SCR, supraclavicular region. ^a^At age 15 days. ^b^HMW adiponectin and CXCL14 assessments were performed in 17 out of 27 girls and 17 out of 23 boys at 4 months and in 22 out of 27 girls and 18 out of 23 boys at 12 months. ^c^The assessment of BAT activity was performed in 22 out of 27 girls and in 18 out of 23 boys at 12 months. *P < 0.05, **P < 0.01, and ***P < 0.001 vs. girls at birth, at age 4 and 12 months and for 0–12-month changes. P-values are adjusted for ponderal index and breastfeeding.

**FIGURE 1:**
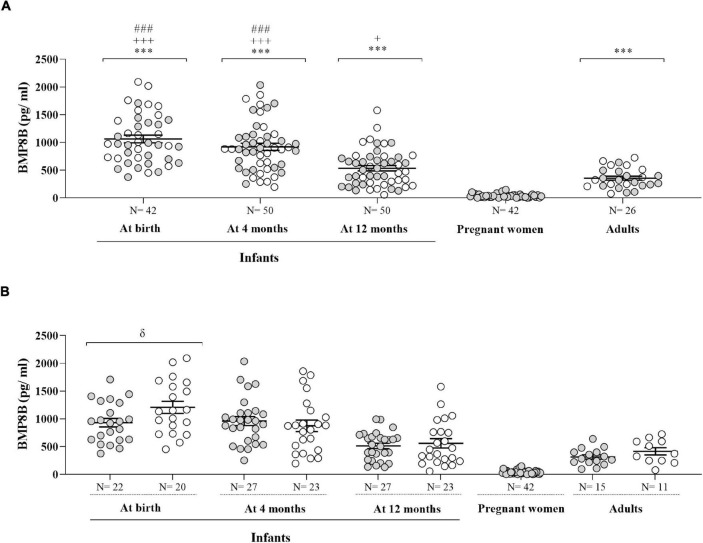
Circulating BMP8B concentrations in human infants at birth and at age 4 and 12 months, in mothers of those infants during the third trimester of pregnancy (gestational age: 35.3 ± 0.39 weeks) and in healthy adult women and men. Data are shown for the two sexes pooled **(A)** and split by sex **(B)**. Gray dots represent girls, pregnant women and healthy adult women, and white dots correspond to boys and to healthy adult men. ^***^P < 0.001 vs. pregnant women; ^+^*P* < 0.05 and ^+++^*P* < 0.001 vs. healthy adults; ^###^*P* < 0.001 vs. 12 months; ^δ^ *P* = 0.04 vs. boys.

Figure 1 depicts circulating BMP8B concentrations in the infants from birth to age 12 months, as well as in the mothers of those infants in the third trimester of gestation and in healthy adults. Over the first year of life, circulating BMP8B levels decreased. When pooling BMP8B concentrations independently of sex, serum BMP8B levels were significantly higher at birth and at age 4 months as compared to age 12 months (*P* < 0.001) and remained higher versus those in healthy adults at all time points. In addition, serum BMP8B concentrations in pregnant women in late gestation were significantly lower than those in healthy adults or in human infants (*P* < 0.001) (Figure 1A). When BMP8B levels were split by sex, boys showed significantly higher concentrations than girls only at birth (*P* = 0.04) (Figure 1B).

**FIGURE 2:**
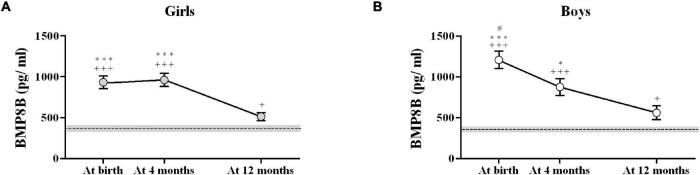
Longitudinal results of circulating BMP8B concentrations in infant girls **(A)** and boys **(B)**. The dotted line depicts the mean values in apparently healthy adults (*N* = 26; 15 women and 11 men); the shaded area represents the standard error (SEM) in those healthy adults. ^#^*P* < 0.05 vs. 4 months; *P < 0.05 and ^***^P < 0.001 vs. 12 months; ^+^*P* < 0.05 and ^+++^*P* < 0.001 vs. healthy adults.

Bone morphogenetic protein-8B levels decreased in a different manner from birth to age 12 months in girls and in boys (Figure 2). While in girls BMP8B levels remained elevated from birth to 4 months and dropped significantly between age 4 and 12 months (Figure 2A), boys experienced a progressive decrease throughout the first year of life (Figure 2B). Indeed, significant differences between BMP8B concentrations at birth and at 4 months were only detected in boys. Interestingly, BMP8B levels were still elevated 36 h after birth in both girls (928 ± 390 pg/ml) and boys (1,656 ± 390 pg/ml) and tended to be higher in boys.

The associations between circulating BMP8B concentrations and clinical, endocrine-metabolic, body composition and BAT activity variables are depicted in Supplementary Table 2. At 4 months, circulating BMP8B levels significantly correlated with bone mineral density (*R* = −0.383; *P* = 0.031) in the entire population, and with serum glucose levels (*R* = 0.467; *P* = 0.042) only in girls. In addition, the decrease in BMP8B levels in the entire population over the first year of life positively correlated with the increase in lean mass (*R* = 0.412; *P* = 0.036).

**FIGURE 3:**
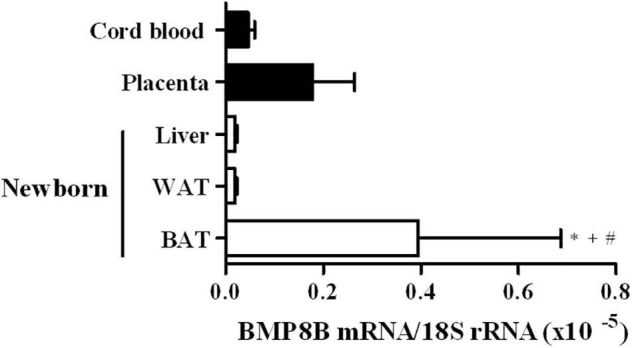
BMP8B expression levels in brown adipose tissue (BAT, *N* = 5), white adipose tissue (WAT, *N* = 5) and liver (*N* = 3) from newborns, and in placenta (*N* = 4) and cord blood cells (*N* = 4). Data are mean ± SEM of relative levels of BMP8B mRNA (BMP8B mRNA/18S rRNA). *P = 0.031 vs. cord blood cells; ^+^*P* = 0.035 vs. liver; ^#^*P* = 0.007 vs. WAT.

The expression levels of BMP8B mRNA in BAT, WAT and liver samples from human neonates and in human placenta and umbilical cord blood cells are shown in Figure 3. BMP8B expression was found to be significantly higher in BAT as compared to WAT, liver and cord blood cells.

## Discussion

The present study constitutes the first one reporting the levels of BMP8B–a brown adipokine involved in thermoregulation and metabolic homeostasis recently identified in experimental studies-across the first year of human development. The highest levels of BMP8B were found at birth, and declined progressively throughout the first year of life, where they were still above adult levels. This pattern is consistent with the especially high requirements of thermoregulation and high BAT activity in neonates early after birth (1). In fact, it is known that maintenance of normothermia at birth is of critical importance, as hypothermia is a challenging and not infrequent condition in newborns (15). Our findings are also consistent with a recent report indicating a positive correlation between BMP8B levels and BAT activity in neonates subjected to thermal stress stimulation in the first 2 days of life (8). Indeed, we found that BMP8B gene expression is the highest in BAT among other neonatal tissues analyzed, which provides the first direct evidence that BAT may be a potential major source of BMP8B in human neonates.

Interestingly, BMP8B levels at birth were higher in boys than in girls, and this trend persisted for at least the next 36 h. This may be in part related to reports of a lower body temperature in boys at birth (16) which may result in enhanced thermal stress, high BAT activation and subsequent BMP8B release. Most reports point to higher BAT activity in adult women than in adult men (17) and there are experimental data indicating that estrogens activate BAT thermogenesis (18) and BMP8B gene expression (19). Along these lines, we disclosed the presence of higher posterior-cervical BAT activity in 1 year-old girls relative to boys (3). In contrast, studies on sex differences in BAT activity in pre-school children have provided heterogeneous conclusions (20–22). Further research is warranted as there are currently no data on sex-related differences in BAT activity in human neonates in the first months of postnatal life.

We did not find significant correlations between BAT activity and BMP8B levels at age 1 year. This may indicate that the reported association between BMP8B levels and inducible BAT activity in 1–2-day-old neonates (8) is restricted to this very early development period characterized by intense thermoregulatory needs and does not happen later in development. It is also possible that the association between BMP8B and BAT activity is evidenced only when BAT activity is determined under acute induction of thermal stress, as in Urisarri et al., (8), and not in basal conditions. Moreover, we did not find significant associations between BMP8B levels and the main auxological, metabolic and endocrine parameters across the first year of life. Indeed, only marginal associations were found between BMP8B levels and each bone mineral density and gains in lean mass in the whole population; these associations could be reminiscent of the role of BMP8B, as member of the bone morphogenetic protein sub-family, in the ossification process (23) and also reflect the effects of gonadal steroids on both lean mass and circulating BMP8B levels (19). Most likely, though, the lack of strong associations could be due to the narrow range of variation in metabolic and endocrine parameters as well as in BAT activity among individuals in the first year of life under healthy conditions. In any case, and according to our findings, variations in BMP8B levels do not appear to be particularly informative of the individual metabolic status and BAT activity in apparently healthy human infants in the first year of life.

An additional noteworthy result in our study is the intense downregulation of BMP8B levels observed during late pregnancy relative to BMP8B levels in non-pregnant women. It has long been known that the thermogenic activity of BAT is decreased at term gestation in rodent models (24, 25). Although no such data on BAT activity are currently available in human pregnancy, low BMP8B levels in pregnant women may reflect reduced BAT thermogenic activity and subsequently reduced BMP8B secretion. On the other hand, the very low BMP8B levels in pregnant women suggest that the substantial expression of BMP8B mRNA in placenta does not result in relevant release of BMP8B to maternal circulation.

The current study has several limitations mostly related to obvious ethical restrictions for the invasiveness of some analytical procedures in healthy neonates. For example, BAT activity data couldn’t be obtained in unrestrained neonates immediately after birth and tissue BAT sampling had to be limited to necropsies from severely ill neonates and was obviously unfeasible in healthy 1-year old infants. However, the longitudinal nature of the study, with extensive information about endocrine-metabolic and body composition parameters to be related to BMP8B levels is a strength of this study, which is to our knowledge the first reporting the outcome of circulating BMP8B levels in humans in the first year of life.

In conclusion, changes in BMP8B levels appear to reflect overall changes in BAT activity in development. However, circulating BMP8B concentrations do not seem to be particularly useful as biomarker of individual variations in the metabolic-endocrine status and BAT activity of healthy infants in the first year of life.

## Author’s Note

CG-B and LI are clinical investigators of CIBERDEM (Centro de Investigación Biomédica en Red de Diabetes y Enfermedades Metabólicas Asociadas, Instituto de Salud Carlos III, Madrid, Spain). AL-B is a clinical investigator of the I3 Fund for Scientific Research (Ministry of Science and Innovation, Spain). FV is an ICREA Academia researcher.

## Data Availability Statement

The original contributions presented in the study are included in the article/Supplementary Material, further inquiries can be directed to the corresponding author/s.

## Ethics Statement

The studies involving human participants were reviewed and approved by the Institutional Review Board of University of Barcelona, Sant Joan de Déu University Hospital. Written informed consent to participate in this study was provided by the participants’ legal guardian/next of kin.

## Author Contributions

CG-B contributed to literature research, design of figures and tables, data collection, analysis, and interpretation, and wrote the manuscript. JV, CP, AG-N, and RC contributed to data collection, analysis, and interpretation. PC contributed to data analysis and interpretation. FZ and AL-B contributed to data interpretation, and reviewed and edited the manuscript. LI and FV contributed to study design, data interpretation, wrote the manuscript, and reviewed and edited the manuscript. All authors contributed to the article and approved the submitted version.

## Conflict of Interest

The authors declare that the research was conducted in the absence of any commercial or financial relationships that could be construed as a potential conflict of interest. The reviewer ML, declared past co-authorship with the authors AG-N, RC, and FV, and the absence of any ongoing collaboration with any of the authors to the handling editor.

## Publisher’s Note

All claims expressed in this article are solely those of the authors and do not necessarily represent those of their affiliated organizations, or those of the publisher, the editors and the reviewers. Any product that may be evaluated in this article, or claim that may be made by its manufacturer, is not guaranteed or endorsed by the publisher.
