# Supplementary figures and images for: Bone Morphogenetic Protein-8B Levels at Birth and in the First Year of Life: Relation to Metabolic-Endocrine Variables and Brown Adipose Tissue Activity
Source: Front Pediatr. 2022 Mar 24;10:869581. doi: 10.3389/fped.2022.869581 (PMC8988030; doi:10.3389/fped.2022.869581)

**Supplemental Figure 1.** Recruitment of the study population

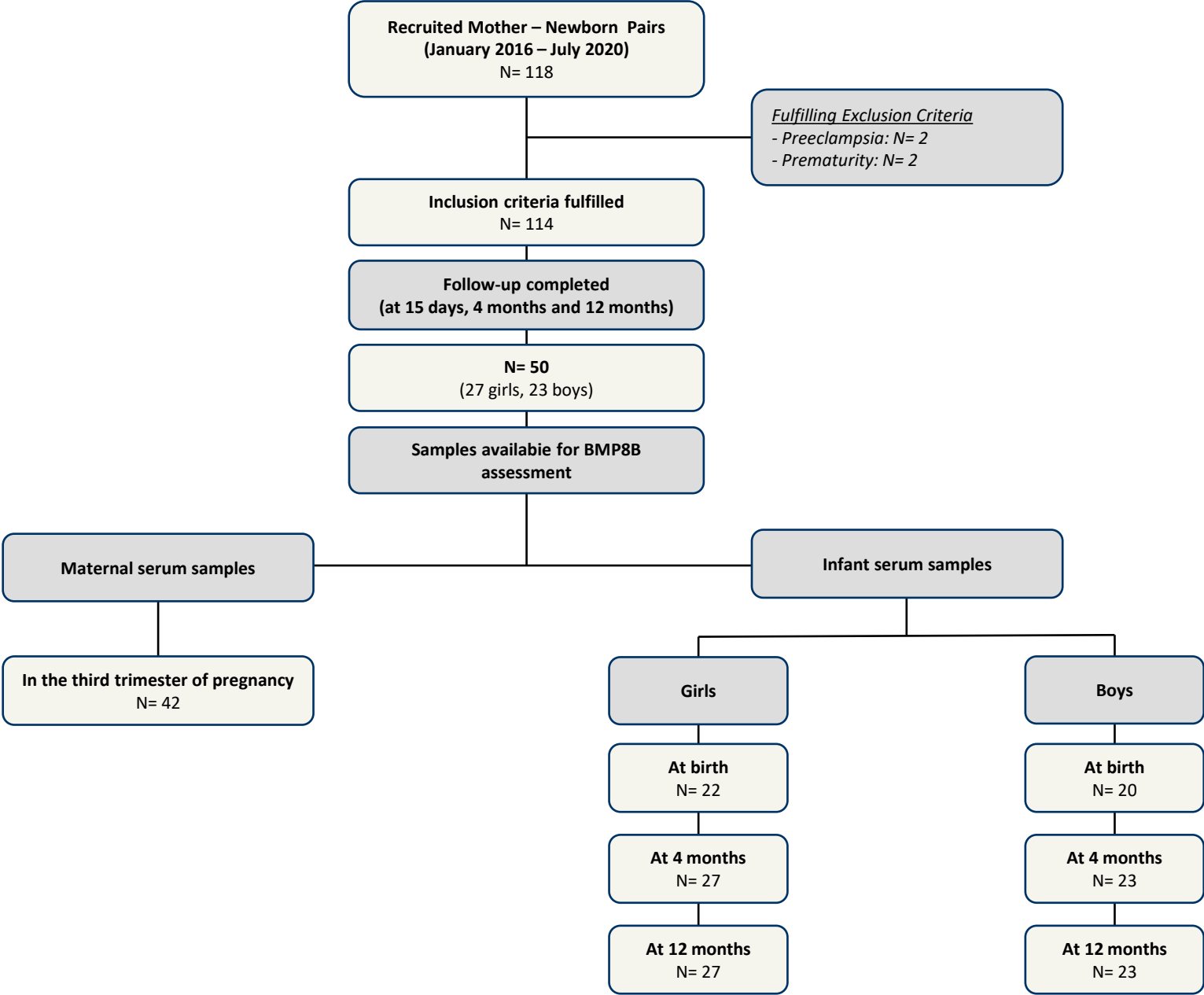

Supplement: Supplementary file 1 [file Image_1.pdf]
