# Supplementary material for: Bone Morphogenetic Protein-8B Levels at Birth and in the First Year of Life: Relation to Metabolic-Endocrine Variables and Brown Adipose Tissue Activity
Source: Front Pediatr. 2022 Mar 24;10:869581. doi: 10.3389/fped.2022.869581 (PMC8988030; doi:10.3389/fped.2022.869581)
Supplement: Supplementary file 2 [file Table_1.DOCX]

**Supplemental Table 1.** Data of newborn subjects included in the study of bone morphogenetic protein 8-B (BMP8B) gene expression.

| **Sample**  **identification** | **Gestational age**  **(weeks)** | **Sex** | **Birth weight**  **(g)** | **Survival**  **(hours)** | **Clinical diagnosis** | **Tissue sample** |
| --- | --- | --- | --- | --- | --- | --- |
| A81 | 28 | F | 1050 | 0 | MM | B, L |
| A87 | 28 | M | 980 | 64 | PA, EOI, | L,W |
| A91 | 30 | M | 830 | 57 | ICH | B,W |
| A98 | 33 | M | 1840 | 10 | LH, RDS | B, L,W |
| A109 | 36 | M | 1860 | 3 | PNO, RA | B, L, W |
| A115 | 32 | M | 2060 | 41 | PH, RDS, ICH, IA, | B, W |

EOI, early onset infection; IA, intrauterine asphyxia; ICH, intracranial hemorrhage; LH, lung hypoplasia; MM, multiple malformations; PA, perinatal asphyxia; PH, pulmonary hemorrhage; PNO, pneumothorax; RA, renal agenesis; RDS, respiratory distress syndrome.

B, dorsal brown adipose tissue; L, liver; W, visceral (omental/ perirenal) white adipose tissue.
