# Supplementary material for: Bone Morphogenetic Protein-8B Levels at Birth and in the First Year of Life: Relation to Metabolic-Endocrine Variables and Brown Adipose Tissue Activity
Source: Front Pediatr. 2022 Mar 24;10:869581. doi: 10.3389/fped.2022.869581 (PMC8988030; doi:10.3389/fped.2022.869581)
Supplement: Supplementary file 3 [file Table_2.DOCX]

**Supplemental Table 2.** Associations between circulating BMP8B levels and clinical, endocrine-metabolic, body composition and BAT activity variables.

| **At birth** | | **All**  **(N= 42)** | | **Girls**  **(N= 22)** | | **Boys**  **(N= 20)** | | **Δ 0-12 months** | | **All**  **(N= 42)** | | **Girls**  **(N= 22)** | | **Boys**  **(N= 20)** | |
| --- | --- | --- | --- | --- | --- | --- | --- | --- | --- | --- | --- | --- | --- | --- | --- |
|  |  | **R** | ***P*** | **R** | ***P*** | **R** | ***P*** |  |  | **R** | ***P*** | **R** | ***P*** | **R** | ***P*** |
| **BMP8B**  **(pg/ ml)** | Weight (kg) | 0.255 | 0.219 | 0.190 | 0.575 | 0.138 | 0.668 | **Δ BMP8B**  **(pg/ ml)** | Δ Weight (kg) | 0.277 | 0.171 | 0.094 | 0.842 | 0.450 | 0.070 |
|  | Weight Z-score | 0.319 | 0.120 | 0.222 | 0.512 | 0.939 | 0.206 |  | Δ Weight Z-score | 0.032 | 0.878 | 0.357 | 0.431 | 0.226 | 0.383 |
|  | Length (cm) | 0.259 | 0.211 | 0.203 | 0.550 | 0.130 | 0.688 |  | Δ Length (cm) | 0.136 | 0.506 | -0.047 | 0.921 | 0.498 | 0.052 |
|  | Length Z-score | 0.315 | 0.125 | 0.224 | 0.508 | 0.334 | 0.289 |  | Δ Length Z-score | 0.077 | 0.709 | 0.118 | 0.801 | 0.398 | 0.114 |
|  | BMI (kg/ m^2^) | 0.235 | 0.257 | 0.192 | 0.572 | 0.085 | 0.792 |  | Δ BMI (kg/ m^2^) | 0.082 | 0.692 | 0.414 | 0.356 | 0.151 | 0.563 |
|  | BMI Z-score | 0.203 | 0.330 | 0.201 | 0.553 | 0.087 | 0.789 |  | Δ BMI Z-score | 0.021 | 0.919 | 0.422 | 0.346 | 0.121 | 0.644 |
|  | BMD (g/cm^2^) | 0.374 | 0.066 | 0.474 | 0141 | 0.048 | 0.187 |  | Δ BMD (g/cm^2^) | -0.024 | 0.906 | 0.051 | 0.914 | 0.047 | 0.859 |
|  | Fat mass (kg) | 0.126 | 0.550 | 0.079 | 0.819 | -0.014 | 0.965 |  | Δ Fat mass (kg) | 0.056 | 0.789 | -0.010 | 0.983 | 0.190 | 0.464 |
|  | Abdominal fat (kg) | 0.132 | 0.531 | 0.103 | 0.764 | 0.002 | 0.995 |  | Δ Abdominal fat (kg) | -0.087 | 0.673 | -0.153 | 0.744 | 0.195 | 0.454 |
|  | Lean mass (kg) | 0.181 | 0.387 | 0.049 | 0.886 | -0.029 | 0.928 |  | Δ Lean mass (kg) | **0.412** | **0.036** | 0.410 | 0.361 | 0.338 | 0.185 |
|  | Fat-to-lean mass ratio | -0.037 | 0.862 | 0.093 | 0.787 | -0.030 | 0.929 |  | Δ Fat-to-lean mass ratio | -0.134 | 0.513 | -0.078 | 0.869 | 0.066 | 0.802 |
| **At 4 months** | | **All**  **(N= 50)** | | **Girls**  **(N= 27)** | | **Boys**  **(N= 23)** | | **At 12 months** | | **All**  **(N= 50)** | | **Girls**  **(N= 27)** | | **Boys**  **(N= 23)** | |
|  |  | **R** | ***P*** | **R** | ***P*** | **R** | ***P*** |  |  | **R** | ***P*** | **R** | ***P*** | **R** | ***P*** |
| **BMP8B**  **(pg/ ml)** | Weight (kg) | -0.244 | 0.179 | -0.209 | 0.493 | -0.261 | 0.311 | **BMP8B**  **(pg/ ml)** | Weight (kg) | 0.296 | 0.089 | -0.010 | 0.976 | 0.458 | 0.062 |
|  | Weight Z-score | -0.281 | 0.119 | -0.215 | 0.482 | -0.258 | 0.318 |  | Weight Z-score | 0.250 | 0.153 | -0.011 | 0.979 | 0.455 | 0.074 |
|  | Length (cm) | -0.229 | 0.207 | -0.170 | 0.579 | -0.260 | 0.314 |  | Length (cm) | 0.326 | 0.060 | -0.030 | 0.926 | 0.431 | 0.076 |
|  | Length Z-score | -0.266 | 0.142 | -0.175 | 0.568 | -0.261 | 0.312 |  | Length Z-score | 0.307 | 0.078 | -0.037 | 0.909 | 0.332 | 0.086 |
|  | BMI (kg/ m^2^) | -0.256 | 0.157 | -0.205 | 0.501 | -0.292 | 0.256 |  | BMI (kg/ m^2^) | 0.293 | 0.093 | 0.009 | 0.978 | 0.424 | 0.063 |
|  | BMI Z-score | -0.281 | 0.119 | -0.196 | 0.522 | -0.244 | 0.345 |  | BMI Z-score | 0.239 | 0.174 | 0.038 | 0.907 | 0.425 | 0.062 |
|  | BMD (g/cm^2^) | **-0.383** | **0.031** | -0.346 | 0.087 | -0.003 | 0.900 |  | BMD (g/cm^2^) | 0.063 | 0.722 | -0.243 | 0.447 | 0.343 | 0.139 |
|  | Fat mass (kg) | -0.253 | 0.162 | -0.189 | 0.536 | -0.361 | 0.154 |  | Fat mass (kg) | 0.122 | 0.491 | -0.144 | 0.723 | 0.288 | 0.218 |
|  | Abdominal fat (kg) | -0.240 | 0.186 | -0.088 | 0.776 | -0.378 | 0.135 |  | Abdominal fat (kg) | 0.026 | 0.884 | -0.238 | 0.455 | 0.220 | 0.352 |
|  | Lean mass (kg) | -0.156 | 0.392 | -0.208 | 0.496 | -0.219 | 0.399 |  | Lean mass (kg) | 0.311 | 0.073 | 0.137 | 0.672 | 0.422 | 0.064 |
|  | Fat-to-lean mass ratio | -0.187 | 0.306 | -0.080 | 0.794 | -0.347 | 0.172 |  | Fat-to-lean mass ratio | -0.046 | 0.796 | -0.119 | 0.712 | 0.099 | 0.679 |
|  | Glucose (mmol/l) | 0.159 | 0.386 | **0.467** | **0.042** | -0.253 | 0.327 |  | Glucose (mmol/l) | -0.256 | 0.130 | -0.246 | 0.441 | -0.289 | 0.216 |
|  | Insulin (pmol/l) | 0.152 | 0.406 | 0.527 | 0.064 | -0.009 | 0.973 |  | Insulin (pmol/l) | -0.013 | 0.944 | -0.365 | 0.243 | 0.413 | 0.070 |
|  | IGF-I (µg/l) | 0.052 | 0.779 | 0.025 | 0.935 | 0.495 | 0.063 |  | IGF-I (µg/l) | -0.158 | 0.372 | -0.344 | 0.273 | 0.145 | 0.542 |
|  | HMW-adip (mg/l) ^†^ | -0.206 | 0.357 | -0.344 | 0.331 | -0.078 | 0.830 |  | HMW-adip (mg/l) ^†^ | -0.208 | 0.319 | -0.064 | 0.829 | -0.547 | 0.127 |
|  | CXCL14 (ng/ml) ^†^ | -0.157 | 0.486 | 0.040 | 0.912 | -0.282 | 0.429 |  | CXCL14 (ng/ ml) ^†^ | 0.162 | 0.440 | 0.083 | 0.778 | 0.466 | 0.206 |
|  |  |  |  |  |  |  |  |  | T_PCR_ (^o^C) ^#^ | -0.208 | 0.364 | 0.066 | 0.846 | -0.348 | 0.398 |
|  |  |  |  |  |  |  |  |  | T_PCR_ – T_SK_ (^o^C) ^#^ | -0.049 | 0.832 | -0.119 | 0.727 | 0.096 | 0.821 |
|  |  |  |  |  |  |  |  |  | Area _PCR_ (px^2^) ^#^ | 0.178 | 0.440 | -0.014 | 0.967 | 0.472 | 0.237 |
|  |  |  |  |  |  |  |  |  | T_SCR_ (^o^C) ^#^ | -0.046 | 0.845 | 0.152 | 0.665 | -0.138 | 0.745 |
|  |  |  |  |  |  |  |  |  | T_SCR_ – T_SK_ (^o^C) ^#^ | 0.026 | 0.911 | 0.120 | 0.726 | 0.005 | 0.991 |
|  |  |  |  |  |  |  |  |  | Area _SCR_ (px^2^) ^#^ | 0.025 | 0.913 | 0.049 | 0.887 | -0.281 | 0.500 |

BMP8B, bone morphogenetic protein 8-B; BMI, body mass index; IGF-I, insulin-like growth factor-I; HMW-adip, high-molecular-weight adiponectin; CXCL14, C-X-C motif chemokine ligand 14; PCR, posterior cervical region; SCR, supraclavicular region.

^†^ HMW adiponectin and CXCL14 assessments were performed in 17 out of 27 girls and 17 out of 23 boys at 4 months and in 22 out of 27 girls and 18 out of 23 boys at 12 months; ^#^the assessment of BAT activity was performed in 22 out of 27 girls and in 18 out of 23 boys at 12 months.

Results are shown as R coefficients and *P* values, adjusted for ponderal index and breastfeeding in multiple regression analysis. Statistically significant values are in bold.
